# From memorization to generalization: fine-tuning large language models for biomedical term-to-identifier normalization

**DOI:** 10.3389/fdgth.2026.1783907

**Published:** 2026-04-02

**Authors:** Suswitha Pericharla, Daniel B. Hier, Tayo Obafemi-Ajayi

**Affiliations:** 1 Computer Science Department, Missouri State University, Springfield, MO, United States; 2 Department of Neurology and Rehabilitation, University of Illinois at Chicago, Chicago, IL, United States; 3 Engineering Program, Missouri State University, Springfield, MO, United States

**Keywords:** biomedical ontologies, fine-tuning, gene ontology, generalization, HGNC, human phenotype ontology, large language models, lexicalization

## Abstract

**Introduction:**

Biomedical data integration requires term-to-identifier normalization, the process of linking natural-language biomedical terms to standardized ontology codes so that extracted concepts become computable and interoperable. Although large language models perform well on clinical text summarization and concept extraction, they remain markedly less accurate at mapping ontology terms to their corresponding identifiers.

**Methods:**

We examined the roles of memorization and generalization in term-to-code mapping across the Human Phenotype Ontology (HPO), the Gene Ontology (GO), and the HGNC gene naming system, including mappings between gene names, lexicalized gene symbols, and arbitrary gene identifiers. Performance was assessed across multiple base models and after task-specific fine-tuning.

**Results:**

Accuracy scaled with model size, with GPT-4o outperforming Llama 3.1 70B and Llama 3.1 8B. Fine-tuning improved forward mappings from term to identifier, with larger gains for GO than for HPO and minimal improvement for gene name-to-HGNC identifier mappings. Generalization to withheld mappings occurred primarily for HGNC gene name-to-gene symbol tasks, whereas fine-tuning on HPO and GO identifiers produced little generalization. Embedding analyses revealed strong semantic alignment between gene names and HGNC gene symbols but no comparable alignment between concept names and identifiers in GO, HPO, or HGNC.

**Conclusions:**

These results suggest that fine-tuning success depends on two interacting factors: popularity and lexicalization. Popularity, a proxy for pretraining exposure to term-identifier pairs, predicted baseline accuracy and the magnitude of memorization gains during fine-tuning, whereas long-tail identifiers remained difficult to consolidate. Lexicalization, the extent to which a symbol functions as a meaningful token in embedding space, enabled generalization and explains why generalization emerged for HGNC gene symbols but not for the arbitrary identifiers used in GO and HPO. Together, these findings provide a predictive framework for identifying when fine-tuning can improve factual term normalization, when gains primarily reflect memorization, and when normalization is likely to fail.

## Introduction

Precision medicine and biomedical data science increasingly depend on transforming free-text mentions of clinical and biological concepts into *computable* representations. A central step is *term-to-identifier normalization*: mapping a natural language term (e.g., tremor) to the standardized code that uniquely represents that concept in a controlled vocabulary or ontology (e.g., HP:0001337 in the Human Phenotype Ontology, HPO). Without this linking step, extracted concepts cannot be reliably joined across datasets, counted, queried, or reused for downstream analytics and machine learning. Although large language models (LLMs) excel at clinical text summarization and concept extraction [1, 2], they perform surprisingly poorly on term → identifier mapping; for example, GPT-4o achieves only about 8% accuracy on HPO term → identifier queries [3]. Bombieri et al. [4] systematically examined the label → ID memorization problem across the Gene Ontology, Uberon, and ICD-10 using zero-shot evaluation of pretrained LLMs, establishing that memorization accuracy correlates strongly with the frequency of concept—identifier co-occurrence on the Web. They reported low accuracy on most term-to-identifier mapping tasks. Building on this evaluation framework, we extend the investigation from pretrained memorization to supervised fine-tuning, treating term–identifier mapping as an acquisition process and experimentally examining how arbitrary associations can be acquired through parameter updates.

When framed as fact retrieval (e.g., “What is the identifier for tremor?”), these failures resemble a long-tail knowledge problem: rare term–identifier pairs are unlikely to be learned robustly from uneven exposure during pretraining. Supervised fine-tuning has been used for knowledge injection to improve *memorization* of such mappings, yet gains can be inconsistent and may not extend beyond trained facts. In this study, we examine which factors govern when fine-tuning succeeds or fails at memorizing ontology term → identifier mappings, and why some long-tail facts remain difficult to acquire even after fine-tuning.

Generative autoregressive LLMs, such as models from the GPT and Llama series, generate text by predicting the next token in a sequence. Despite being optimized for language modeling, they exhibit emergent abilities in factual recall and are increasingly used as repositories of information [5–9]. However, factual use remains imperfect: models may fail to retrieve known facts or produce incorrect ones (hallucinations) [10]. Wang et al. [11] distinguish fact-based queries (e.g., “What is the HPO identifier for tremor?”) from skill-based queries (e.g., “Summarize this physician note”), a distinction that is central to ontology normalization. Retrieval-augmented generation (RAG) and supervised fine-tuning have been proposed to address factual gaps and improve reliability [12–15]. Fine-tuning can inject knowledge [16], but its effects can be inconsistent and may induce degradation of previously correct knowledge [17]. Moreover, fine-tuning is most effective when target facts are already partially represented in the base model, whereas genuinely novel or extremely rare facts are harder to acquire through fine-tuning alone [9, 18]. Related work has also emphasized that fine-tuning may overfit through rote memorization, generalize poorly to unseen facts, and exhibit directional biases such as the *reversal curse* [7, 11, 19–25].

A key distinction among biomedical terminologies is whether the code associated with a term is an *arbitrary identifier* or a *lexicalized symbol*. As summarized in Table 1, some ontologies—such as HPO and the Gene Ontology (GO)—assign each concept an arbitrary identifier (e.g., HP:0001250 for Seizure). These identifiers function as semantically opaque codes and intentionally provide no surface cues about meaning. In contrast, the HUGO Gene Nomenclature Committee (HGNC) provides both a lexicalized gene symbol (e.g., TP53 for tumor protein p53) and an arbitrary gene ID (e.g., HGNC:11998) for each gene [26]. Gene symbols are intentionally mnemonic and are designed to behave like meaningful tokens, whereas gene IDs, like ontology identifiers, are semantically opaque.

**Table 1:** Examples of arbitrary and lexicalized biomedical codes.

Terminology	Term	Code	Type	Category
GO	Nucleus	GO:0005634	ID	Arbitrary
HPO	Tremor	HP:0001337	ID	Arbitrary
HGNC	Tumor protein p53	TP53	Symbol	Lexicalized
HGNC	Tumor protein p53	HGNC:11998	ID	Arbitrary

GO, Gene Ontology; HPO, Human Phenotype Ontology; HGNC, HUGO Gene Nomenclature Committee.

Arbitrary identifiers do not resemble the linked term. Lexicalized symbols are designed to evoke the linked term. Gene names are linked to both arbitrary identifiers and lexicalized gene symbols.

This distinction suggests a mechanistic hypothesis about fine-tuning outcomes. When LLMs are fine-tuned on arbitrary identifiers, they must rely primarily on rote memorization of token sequences because the identifiers carry little intrinsic semantic signal. Lexicalized symbols, by contrast, can participate in the model’s preexisting semantic embedding space and may align naturally with associated biomedical terms. Therefore, fine-tuning on lexicalized gene symbols should better support generalization to unseen term–symbol pairs, whereas fine-tuning on arbitrary identifiers should be constrained largely to memorization.

These theoretical distinctions motivated the design of our experiments. For HPO and GO, which provide only arbitrary identifiers for each concept, we fine-tuned two models for each ontology: (*term* → *identifier*) and (*identifier* → *term*). For HGNC, which assigns both an arbitrary gene identifier and a lexicalized gene symbol to each gene, we fine-tuned four models: (*term* → *identifier*), (*identifier* → *term*), (*term* → *symbol*), and (*symbol* → *term*). Prior work has demonstrated that the frequency of a fact in the pretraining corpus (*fact popularity*) predicts whether an LLM retrieves that fact [5]. We previously showed that fact popularity predicts LLM performance on ontology term-to-identifier mapping tasks [3] and constrains whether such mappings can be learned during fine-tuning [18]. Building on these insights, we constructed frequency-balanced datasets for all three terminologies to isolate the relative influence of identifier popularity and identifier lexicalization on fine-tuning performance. Across the three terminologies (HPO, GO, and HGNC), several empirical findings emerged:

1. *Memorization*: Mapping accuracy varied systematically by fine-tuning task (HGNC term → symbol ≫ GO term → ID ≫ HPO term → ID ≫ HGNC term → HGNC ID).
2. *Generalization*: Generalization from trained (seen) terms to withheld (unseen) terms occurred only for the HGNC term → symbol and symbol → term tasks.
3. *Directionality*: Across terminologies, mapping accuracy after fine-tuning was consistently higher in the forward direction (term → identifier) than in the reverse direction (identifier → term).
4. *Degradation*: When the base model possessed correct term → identifier mappings, some of this knowledge degraded for withheld (unseen) terms during fine-tuning.

We interpret these results through two vantage points: *lexicalization* and *popularity*. Lexicalization refers to the extent to which symbols and identifiers carry meaningful semantic signals within the model parameters [27]. Popularity refers to the extent to which the prevalence of terms and identifiers in the pretraining corpus influences the salience and recoverability of facts within those parameters [5].

We study fine-tuning not as a proposed replacement for retrieval-augmented generation or algorithmic lookup methods—which remain more reliable for production-grade term normalization [13, 14]—but as a controlled experimental framework for investigating the conditions under which LLMs acquire arbitrary factual associations through parameter updates. Term–identifier pairs are an ideal testbed for this purpose precisely because correct retrieval cannot be inferred from surface form and must be memorized, making these mappings a sensitive probe of LLM fact acquisition mechanisms.

The remainder of this article is organized as follows. In *Methods*, we describe dataset construction, define the mapping tasks, and explain how fine-tuned models were assessed for accuracy, memorization, generalization, and knowledge degradation. In *Results*, we report performance in both mapping directions across HPO, GO, and HGNC. In *Discussion*, we argue that lexicalization and popularity jointly explain differences in model performance across terminologies, mapping directions, and outcomes, and we conclude with study limitations, future directions, and implications for fine-tuning LLMs on ontology term-to-identifier normalization.

## Methods

**Figure 1:**
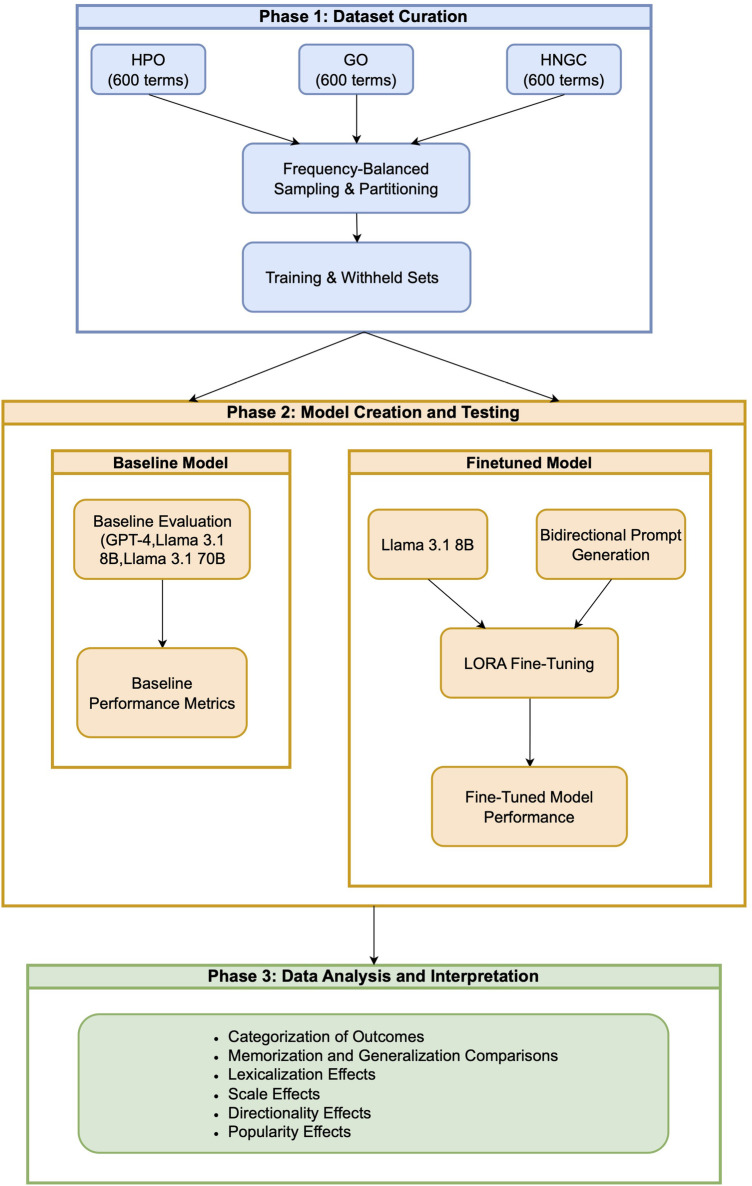
Overview of Methods. Phase 1: frequency-balanced train and validation test sets were created from three biomedical terminologies. Phase 2: Fine-tuned models were created for each terminology and performance compared to baseline models. Phase 3: Gains from fine-tuning were attributed to either memorization or generalization. Effects of scale, popularity, directionality, and lexicalization were assessed.

We evaluated the ability of LLMs to link biomedical terms to their corresponding identifiers across three widely used terminologies: the Human Phenotype Ontology (HPO) [28], the Gene Ontology (GO) [29], and gene names from the HUGO Gene Nomenclature Committee (HGNC) [30]. These resources differ fundamentally in the degree to which their identifiers are lexicalized, providing a natural testbed for examining how LLMs learn term–identifier mappings. The overall research workflow is summarized in Figure 1.

### Datasets

The Human Phenotype Ontology contains approximately 18,800 terms that describe phenotypic abnormalities observed in human disease. HPO is widely used to annotate the clinical features of rare disorders, with more than 200,000 phenotype annotations across approximately 8,000 human diseases [31]. Each concept is associated with a semantically arbitrary identifier (Table 1).

The Gene Ontology comprises 43,303 concepts organized into three hierarchies (biological processes, molecular functions, and cellular components). Across species, GO includes more than 9 million curated annotations [32] that link gene products to GO concepts. Like HPO, GO assigns each concept a semantically arbitrary identifier.

The HUGO Gene Nomenclature Committee has approved approximately 44,400 gene symbols, of which 19,250 correspond to protein-coding genes. Each gene has a textual name (*tumor protein p53*), a lexicalized symbol (e.g., TP53), and an arbitrary identifier (e.g., HGNC:11998). Protein-coding genes are linked to entries in UniProtKB [33, 34]. In contrast to HPO and GO identifiers, HGNC gene symbols are designed to evoke the gene name or function [26].

These three terminologies therefore differ along two key dimensions: (1) lexicalization of their codes and (2) the frequency with which those codes appear in the biomedical literature. HPO and GO provide only arbitrary, non-lexicalized identifiers, whereas HGNC uniquely provides both arbitrary identifiers and mnemonic gene symbols for the same biological entity. This design allows a direct within-terminology comparison of lexicalized and non-lexicalized codes. In addition, identifier and symbol popularity varies substantially across terminologies, enabling examination of the independent and interacting effects of lexicalization and popularity. Together, these properties provide a robust testbed for assessing memorization and generalization of term–code mappings during fine-tuning.

### Sampling strategy

Biomedical terminologies exhibit a pronounced *long-tail* distribution consistent with Zipf’s law [35]: a small number of head terms appear frequently in the literature, whereas the vast majority occur rarely. LLMs consistently struggle with long-tail items because such terms receive little or no exposure during pretraining [18, 36–38]. Identifier usage counts were retrieved from PubMed Central (PMC) full-text database via the PMC API [39]. For each terminology, identifiers were ranked by their corpus frequency, partitioned into twenty equal-sized bins by rank, and thirty term–identifier pairs were randomly sampled from each bin. Because biomedical terms frequently appear without their identifiers, identifier frequency is a more reliable measure of long-tail behavior than term frequency alone [3, 18]. As noted previously, 40%–50% of HPO and GO identifiers never appear in the biomedical literature as annotations [40]. Such identifiers were excluded from sampling because they provide no measurable evidence of use.

Since it is infeasible to fine-tune on every term in a large ontology, we constructed a frequency-balanced dataset for each terminology (HPO, GO, and HGNC) consisting of 600 terms sampled proportionally from the head, body, and tail of the usage distribution [3], after ranking terms according to the frequency of the ID or gene symbol in the PMC. For GO, which spans three major hierarchies (cellular component, biological process, and molecular function), we sampled 200 term–identifier pairs from each hierarchy to preserve structural balance. These curated datasets formed the foundation for the baseline model evaluation prior to fine-tuning.

### Baseline evaluation

Baseline performance was evaluated using unmodified Llama 3.1 models (8B and 70B parameters) [41] and GPT-4o [42]. All models were tested on the same term–identifier datasets using identical prompt templates (Table 2), allowing for a direct comparison of pretrained knowledge across model families. This baseline assessment quantifies the biomedical term–identifier mappings that are accessible to each model without any task-specific training and provides a reference point for evaluating subsequent gains from fine-tuning.

**Table 2:** Prompt templates for term–identifier mapping fine-tuning.

Prompt	Template
1	What is the [ONTOLOGY] identifier for the [ONTOLOGY] term [TERM]?
2	The [ONTOLOGY] term [TERM] has what [ONTOLOGY] identifier?
	Respond only with the [ONTOLOGY] identifier.
3	Provide the [ONTOLOGY] identifier for: [TERM]
4	What is the ontology identifier for [TERM] in [ONTOLOGY]?
5	Return only the [ONTOLOGY] identifier for the term: [TERM]

Each term–identifier pair was expanded into five distinct prompts to capture variation in phrasing. [ONTOLOGY] is the placeholder for the terminology, and [TERM] is the placeholder for the term being probed. Reverse mapping prompts mirrored the forward prompts, substituting identifiers for terms. For example, in the forward direction, a sample prompt could be *What is the HPO identifier for the HPO term ataxia?*.

All models were queried using greedy decoding (temperature = 0) to ensure deterministic outputs and eliminate response variability across repeated queries. Because greedy decoding produces deterministic outputs, a single evaluation pass per instance was deemed sufficient.

Performance was measured in both mapping directions (*term* → *identifier* and *identifier* → *term*) for all three terminologies (HPO, GO, and HGNC). Mapping accuracy was quantified using top-1 accuracy (hits@1), where a prediction was scored as correct only if the model’s top-ranked output exactly matched the ground-truth identifier or term. These baseline results establish the level of intrinsic biomedical knowledge acquired during pretraining and set the stage for evaluating whether parameter-efficient fine-tuning can enable the acquisition of domain-specific mappings without full model retraining.

### Parameter-efficient fine-tuning

For each terminology (HPO, GO, and HGNC), we constructed frequency-balanced training sets of 600 term–identifier pairs and corresponding withheld test sets of 600 unseen term–identifier pairs. Note, the list of the training and testing terms are available on our GitHub repository.^1^ Each training pair was expanded into five distinct natural-language prompt templates (Table 2) to provide lexical variety; reverse-mapping prompts mirrored the forward prompts by substituting identifiers for terms. This yielded 3,000 training prompts per terminology per mapping direction. No prompt engineering or prompt optimization was performed; the five templates were fixed a priori and applied uniformly across all terminologies and mapping directions.

Fine-tuning was performed using the Llama-3.1 8B Instruct model through the Together AI supervised fine-tuning API (https://www.together.ai/) on shared cloud infrastructure. Parameter-efficient adaptation used Low-Rank Adaptation (LoRA; rank r=64, scaling α=128) [43] applied to all linear transformer layers. Supervised fine-tuning employed a learning rate of $1\times 10^{-5}$ with a cosine decay scheduler (no warm-up, cycle length = 0.5), batch size 32, maximum gradient norm 1, and no weight decay. Training proceeded for 20 epochs, yielding approximately 1,875 gradient updates per model (3,000 examples × 20 epochs ÷ batch size 32). Models were evaluated at epochs 5, 10, 15, and 20; final model selection used the epoch at which accuracy gains plateaued. Eight models were fine-tuned in total (HPO ×2, GO ×2, HGNC ×4). Wall-clock time varied with platform resource availability; the approximate API cost per fine-tuning run was $0.65 USD based on Together AI’s token-based pricing at the time of experimentation.

Fine-tuning was restricted to Llama 3.1 8B for both practical and scientific reasons. Larger models (e.g., 70B-parameter scale) incur substantially higher fine-tuning costs and were not the focus of this study. Scientifically, the 8B model represents a well-characterized architecture where memorization gains and generalization failures are both observable without ceiling effects; the 70B and GPT-4o baselines establish that scale alone does not resolve the long-tail mapping problem [5].

### Evaluation framework

Fine-tuning performance was assessed by comparing model predictions before and after fine-tuning for each test instance (term–identifier, identifier–term, term–symbol, or symbol–term). Let i index the test instances. For the i-th instance, we define binary indicators $B_{i}$ and $F_{i}$ representing the correctness of the baseline and fine-tuned models, respectively, where 1 indicates a correct prediction and 0, an incorrect prediction. These variables allow us to formally define the outcome of fine-tuning for each instance. Each instance was assigned to one of four mutually exclusive outcome categories:

1. *Gainer*: incorrect at baseline but correct after fine-tuning, indicating that the model successfully acquired the mapping (i.e., $B_{i}=0\wedge F_{i}=1$).
2. *Loser*: correct at baseline but incorrect after fine-tuning, indicating degradation or overwriting of previously encoded knowledge (i.e., $B_{i}=1\wedge F_{i}=0$).
3. *Continuing Correct*: correct at baseline and correct after fine-tuning, indicating preservation of existing knowledge (i.e., $B_{i}=1\wedge F_{i}=1$).
4. *Continuing Incorrect*: incorrect at baseline and incorrect after fine-tuning, indicating no improvement (i.e., $B_{i}=0\wedge F_{i}=0$).

**Figure 2:**
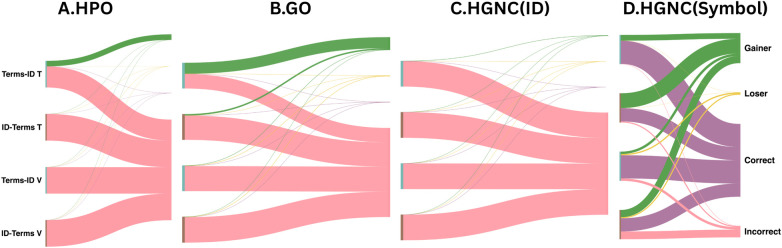
Sankey diagrams illustrating fine-tuning outcomes across HPO, GO, and HGNC terminologies and tasks (term-to-identifier and identifier-to-term under training (T) and validation (V) conditions. Color flows show transition after fine-tuning: purple = continuing correct, orange = green (newly correct with fine-tuning), pink = continuing incorrect after fine-tuning, and yellow = loser (degraded by fine-tuning). Panel **(A)** (HPO) shows the dominance of pink flows (continuing to be incorrect), reflecting the long tail of low-popularity identifiers that remain unmapped after fine-tuning. Panel **(B)** (GO) exhibits stronger green flows, consistent with effective memorization of trained terms. Panel **(C)** (HGNC ID) displays extensive pink flows (continuing incorrect), indicating minimal memorization of HGNC gene-ID pairs. Panel **(D)** shows for HGNC gene-symbols pairs strong green flows (gainers due to fine-tuning) and strong purple flows (continuing correct). Minimal losses due to degradation (yellow flows) are shown.

This four-way classification distinguishes gains, losses, and stable outcomes on a per-instance basis, providing a comprehensive view of how fine-tuning affects knowledge acquisition across the three terminologies. We report the total count in each category and visualize these transitions using Sankey flow diagrams (Figure 2).

### Memorization compared to generalization

In traditional machine learning, memorization and generalization are defined with respect to training, validation, and test sets. Memorization is defined traditionally as the verbatim reproduction of examples from the training set when the model is queried. Generalization is traditionally defined as accurate performance of the model on withheld test items that resemble training examples but that were not seen during training. Following recent work on factual memorization in LLMs [11, 24, 44, 45], we adopt definitions based on whether a term was *seen* during fine-tuning rather than whether it belonged to a training or validation split. We therefore operationalize memorization and generalization as follows:

*Memorization*: the improvement in accuracy on *seen* term–identifier pairs (i.e., pairs included in the fine-tuning training dataset). Let $N_{\text{seen}}$ denote the total number of evaluated seen instances and $G_{\text{seen}}$ the number of instances that were incorrect at baseline but correct after fine-tuning (i.e., gainers). Memorization reflects the extent to which fine-tuning enables the model to correctly retrieve mappings that it was directly exposed to during training (Equation 1).

$$\text{Memorization}\;(\%)=\frac{G_{\text{seen}}}{N_{\text{seen}}}\times 100$$ (1)

*Generalization*: the improvement in accuracy on *unseen* term–identifier pairs (i.e., pairs withheld from fine-tuning). Let $N_{\text{unseen}}$ denote the total number of evaluated unseen instances and $G_{\text{unseen}}$ the number of instances that were incorrect at baseline but correct after fine-tuning (i.e., gainers) (Equation 2). Importantly, these unseen pairs resemble the seen (training) pairs and are drawn from the same ontology.

$$\text{Generalization}\;(\%)=\frac{G_{\text{unseen}}}{N_{\text{unseen}}}\times 100$$ (2)

For Equations 1, 2: $N_{\text{seen}}$ and $N_{\text{unseen}}$ denote the number of evaluated instances in the seen and unseen sets, respectively, and $G_{\text{seen}}$ and $G_{\text{unseen}}$ denote the number of gainers (i.e., instances incorrect at baseline but correct after fine-tuning).

Generalization reflects whether the model can apply what it learned during fine-tuning to mappings that were not encountered during fine-tuning. These definitions align with recent studies showing that improvements on seen facts correspond to rote memorization, whereas improvements on unseen facts reflect generalization beyond the fine-tuning set [45]. Here, *generalization* refers specifically to improvement on withheld term–identifier pairs drawn from the same ontology as the fine-tuning training data. We do not test whether the model generalizes to new ontologies or to new tasks.

### Lexicalization

To understand why some terminologies support generalization while others do not, we examined the degree of *lexicalization*. Here, lexicalization refers to the extent to which an identifier carries semantic information that evokes its associated term. Lexicalized gene symbols typically share semantic overlap with their gene names (e.g., TP53 ↔ tumor protein p53), whereas arbitrary identifiers(e.g., HP:0001250 → *seizure*) do not. For lexicalization analyses, embeddings were derived from the Llama 3.1 8B base model (meta-llama/Meta-Llama-3-8B). For each term, identifier, and gene symbol, we applied the model’s native tokenizer, ran a single forward pass with hidden states returned, and extracted the final-layer hidden state for all tokens. These token-level vectors were then mean-pooled to yield a single 4,096—dimensional embedding per string.

For each true term–identifier, term–symbol, and symbol–identifier pair, we computed a delta value (Δ) defined as the cosine similarity of the matching pair minus the mean cosine similarity across all non-matching pairs. Larger Δ values reflect stronger semantic alignment and were interpreted as evidence of lexicalization in the embedding space. As a complementary analysis to assess semantic alignment between terms and identifiers, all embedding vectors (n=600 per terminology) were projected into two dimensions using principal component analysis (PCA). This visualization tested whether LLM embeddings organize terms, identifiers, and symbols within a shared geometric manifold and whether lexicalized symbols cluster more closely with their associated terms than arbitrary identifiers do.

### Popularity

The frequency with which a fact appears in an LLM’s pretraining corpus—referred to as its *popularity*—has been shown to predict both its retrievability and its ease of consolidation during fine-tuning [5, 18, 23]. Because the exact frequencies of individual facts in the pretraining corpus are unknown, we approximate popularity using the following empirical proxies:

1. *Identifier frequency in biomedical text:* counts of HPO identifiers, GO identifiers, HGNC gene identifiers, and HGNC gene symbols appearing in PubMed Central (PMC) full-text articles [39].
2. *Term frequency in biomedical text:* counts of HPO terms, GO terms, and HGNC gene names in PMC.
3. *Annotation frequency in curated resources:* counts of HPO terms used in disease annotation [31], GO cellular component terms used in functional annotation [32], and UniProtKB protein annotations associated with HGNC gene names [34].

These popularity measures approximate the likelihood that a model encountered a given term–identifier association during pretraining. To reduce extreme skewness in the long-tail distributions, we applied Laplace smoothing (adding one to each count) followed by $\log_{10}$ transformation. The transformed popularity variables were analyzed using two-way analyses of variance (ANOVA) with *Terminology* (HPO, GO, HGNC) and *Correctness* (Match = 0 or 1) as fixed factors. When a significant main effect or interactions were detected, pairwise differences among terminologies were evaluated using the Games–Howell post hoc test, which is robust to unequal variances and unequal sample sizes.

## Results

We evaluated baseline performance across three models (Llama 3.1 8B, Llama 3.1 70B, and GPT-4o) on all three terminologies and in both mapping directions. The fine-tuning experiments used the Llama 3.1 8B model exclusively.

### Baseline performance

Table 3 presents baseline accuracy for all models and terminologies. GPT-4o achieved the highest baseline accuracy in the forward direction (term → identifier) , followed by Llama 3.1 70B and Llama 3.1 8B across all mappings. In the forward direction of mapping, the HGNC term → symbol mappings were the highest accuracies across all LLMs (79.5%–98.7%), the GO mappings were (1.0%–33.3%), the HPO mappings were lower (0.5%–8.8%), and the HGNC term → identifier mappings were the lowest (0.0%–8.5%).

**Table 3:** Baseline and fine-tuned performance across the biomedical terminologies and mapping directions.

Mapping	Llama 8B	Llama 8B FT	Δ FT	Llama 70B	GPT-4o
HPO Identifier → Term	0.0	0.0	0.0	0.0	0.0
HPO Term → Identifier	0.5	21.2	+20.7	5.0	8.8
GO Identifier → Term	0.2	5.8	+5.6	3.8	17.0
GO Term → Identifier	1.0	43.7	+42.7	13.5	33.3
HGNC Term → Identifier	0.0	1.2	+1.2	2.0	8.5
HGNC Identifier → Term	0.0	0.3	+0.3	0.0	0.3
HGNC Symbol → Term	45.8	96.0	+50.2	80.5	89.8
HGNC Term → Symbol	79.5	98.8	+19.3	95.0	98.7

Accuracy values are percentages. Δ FT is the calculated performance gain from fine-tuning Llama 3.1 8B. All models were evaluated on both training and validation terms.

### Effect of fine-tuning on accuracy

Fine-tuning effects for Llama 3.1 8B varied substantially by terminology (Table 3). Accuracy gains (ΔFT) were minimal for the HGNC term → ID mapping but stronger for the HPO term → identifier, GO term → identifier, and HGNC term → symbol mappings. After fine-tuning, the performance of the Llama 3.1 8B model exceeded that of the baseline Llama 3.1 70B and GPT-4o models on the HPO term → identifier, GO term → identifier, and HGNC term → symbol tasks. Across all models and terminologies, term → identifier accuracy exceeded identifier → term accuracy, a pattern observed in both baseline and fine-tuned conditions.

### Knowledge gains and losses following fine-tuning

Table 4 classifies each mapping outcome after fine-tuning into four mutually exclusive categories: *Gainer*, *Loser*, *Continuing Correct*, and *Continuing Incorrect*. Results are reported separately for trained terms (seen during fine-tuning) and withheld terms (unseen during training). These outcomes are also visualized as Sankey flows (Figure 2), which illustrate the movement of mappings between correct and incorrect states following fine-tuning. The Sankey flows highlight striking differences across terminologies. *Gainer* flows (green), representing mappings corrected by fine-tuning, are largest for the HGNC term → symbol task, substantial for GO term → ID mappings, smaller for HPO term → ID mappings, and minimal for the HGNC term → ID task. In contrast, *Continuing Correct* flows (purple) are dominated by HGNC term → symbol mappings, reflecting the relatively high baseline accuracy of this lexicalized mapping task. By comparison, mappings involving numeric identifiers show extensive *Continuing Incorrect* flows (pink), indicating that most errors persist after fine-tuning. This pattern is particularly evident for HPO and GO identifier mappings, where the lack of a lexicalized structure limits the model’s ability to learn or generalize these associations.

**Table 4:** Bidirectional assessment of fine-tuning effectiveness on biomedical mapping.

Terminology	Direction	Category	Withheld %	Trained %
			(Unseen)	(Seen)
HPO (ID)	ID → Term	Gainer	0.0	0.0
		Loser	0.0	0.0
		Continuing correct	0.0	0.0
		Continuing incorrect	100.0	100.0
	Term → ID	Gainer	0.2	20.7
		Loser	0.0	0.0
		Continuing correct	0.2	0.5
		Continuing incorrect	99.0	78.8
GO (ID)	ID → Term	Gainer	0.2	5.7
		Loser	1.3	0.0
		Continuing correct	0.5	0.2
		Continuing incorrect	98.7	94.2
	Term → ID	Gainer	1.2	42.8
		Loser	1.3	0.2
		Continuing correct	0.3	0.8
		Continuing incorrect	97.2	56.2
HGNC (ID)	ID → Term	Gainer	0.5	0.3
		Loser	0.0	0.0
		Continuing correct	0.0	0.0
		Continuing incorrect	99.5	99.7
	Term → ID	Gainer	0.5	1.2
		Loser	0.0	0.0
		Continuing correct	0.0	0.0
		Continuing incorrect	99.5	98.8
HGNC (Symbol)	Symbol → Term	Gainer	24.7	50.5
		Loser	3.7	0.3
		Continuing correct	45.2	45.5
		Continuing incorrect	26.5	3.7
	Term → Symbol	Gainer	8.2	19.5
		Loser	4.3	0.2
		Continuing correct	79.2	79.3
		Continuing incorrect	8.3	1.0

Assessment of fine-tuning effectiveness on biomedical bidirectional mapping performance (Training: 600 pairs per terminology; Withheld: 600 pairs matched for popularity and not seen during training). Results are separated into four outcome categories (defined in Methods) and reported for both trained (seen) and withheld (unseen) terms.

Baseline mapping performance varied widely across tasks prior to fine-tuning. Mappings involving lexicalized identifiers (e.g., HGNC term → symbol) exhibited relatively high baseline accuracy, with approximately 79% of mappings remaining correct (*Continuing Correct*). In contrast, mappings involving arbitrary numeric identifiers (e.g., GO term → GO ID or HPO term → HPO ID) showed near-zero baseline accuracy, reflecting the absence of lexical cues linking ontology terms to their numeric identifiers. Similarly low baseline performance was observed for HGNC term → ID mappings, where the identifiers are likewise non-lexicalized.

Fine-tuning produced markedly different effects across these tasks. For the HPO model, almost all gains (20.7%) occurred in the forward direction (HPO term → HPO ID) and primarily among trained pairs, indicating strong memorization but essentially no generalization to withheld terms (0.2%). A similar pattern was observed for the GO model, where substantial gains were observed on trained pairs (42.8%) but again with minimal generalization (1.2%). In contrast, mappings involving HGNC gene symbols and gene names showed substantially stronger performance. The HGNC term ↔ symbol models exhibited gains in both directions and across both seen and unseen terms, reflecting the strong lexical relationship between gene names and their symbols.

To summarize these effects more directly, Table 5 reports derived performance metrics calculated from the outcome categories in Table 4. These metrics include memorization (gains on trained terms), generalization (gains on withheld terms), degradation (losses following fine-tuning), and overall post–fine-tuning accuracy. Among the evaluated tasks, the HGNC term → symbol model achieved the highest overall accuracy (98.6%). This performance was supported by moderate memorization (19.5%) and measurable generalization (8.2%), with limited degradation (4.3%). The reverse direction (symbol → term) showed larger memorization gains (50.5%) and stronger generalization (24.7%), although final accuracy was slightly lower (95.7%). By contrast, mappings involving HGNC numeric identifiers remained difficult for the model. For the HGNC term → ID task, both memorization (1.2%) and generalization (0.5%) were minimal, and similar results were observed in the reverse direction (ID → term). These results reinforce the broader pattern observed across ontologies: fine-tuning readily strengthens mappings supported by lexical cues but provides little improvement for mappings involving arbitrary numeric identifiers.

**Table 5:** Derived performance metrics for fine-tuned Llama 3.1 8B across the three biomedical terminologies.

Task	Memorized	Generalized	Degraded	Accuracy
HPO Identifier → Term	0.0	0.0	0.0	0.0
HPO Term → Identifier	20.7	0.2	0.0	20.2
GO Identifier → Term	5.7	0.2	1.3	5.9
GO Term → Identifier	42.8	1.2	1.3	44.4
HGNC ID → Term	0.3	0.5	0.0	0.3
HGNC Term → ID	1.2	0.5	0.0	1.2
HGNC Symbol → Term	50.5	24.7	3.7	95.7
HGNC Term → Symbol	19.5	8.2	4.3	98.6

All values are percentages.

Accuracy = Correct + Gainer − Loser on trained terms;

Memorized = gains on trained terms;

Generalized = gains on withheld terms;

Degraded = losses on withheld terms. Values derived from Table 4.

### Lexicalization

**Figure 3:**
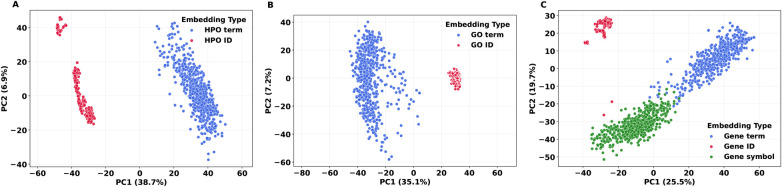
Two-dimensional PCA projections of the 4096-dimensional embeddings generated by Llama 3.1 8B for 600 HPO terms, 600 GO terms, and 600 HGNC gene terms. Panel **(A)** (HPO) and Panel **(B)** (GO) show that identifier strings (red) form compact clusters that are well separated from their corresponding ontology terms (blue), indicating that ontology identifiers are not lexicalized in the model’s embedding space. In Panel **(C)**, HGNC gene symbols (green) and gene names (blue) occupy overlapping regions of the embedding space, indicating strong lexicalization of gene symbols.

To assess whether Llama 3.1 embeddings encode semantic alignment between biomedical terms and their identifiers, we examined both geometric structure and cosine similarity behavior. PCA projection of the 4,096-dimensional embeddings (Figure 3) revealed clear differences across terminologies. HGNC gene names and gene symbols occupied overlapping regions of the embedding space, suggesting semantic alignment. In contrast, HPO and GO identifiers formed clusters that were spatially separated from their corresponding terms, consistent with their design as arbitrary, non-lexicalized machine codes. Like the HPO identifiers and the GO identifiers, the HGNC gene identifier did not show semantic alignment with the HGNC gene names.

**Figure 4:**
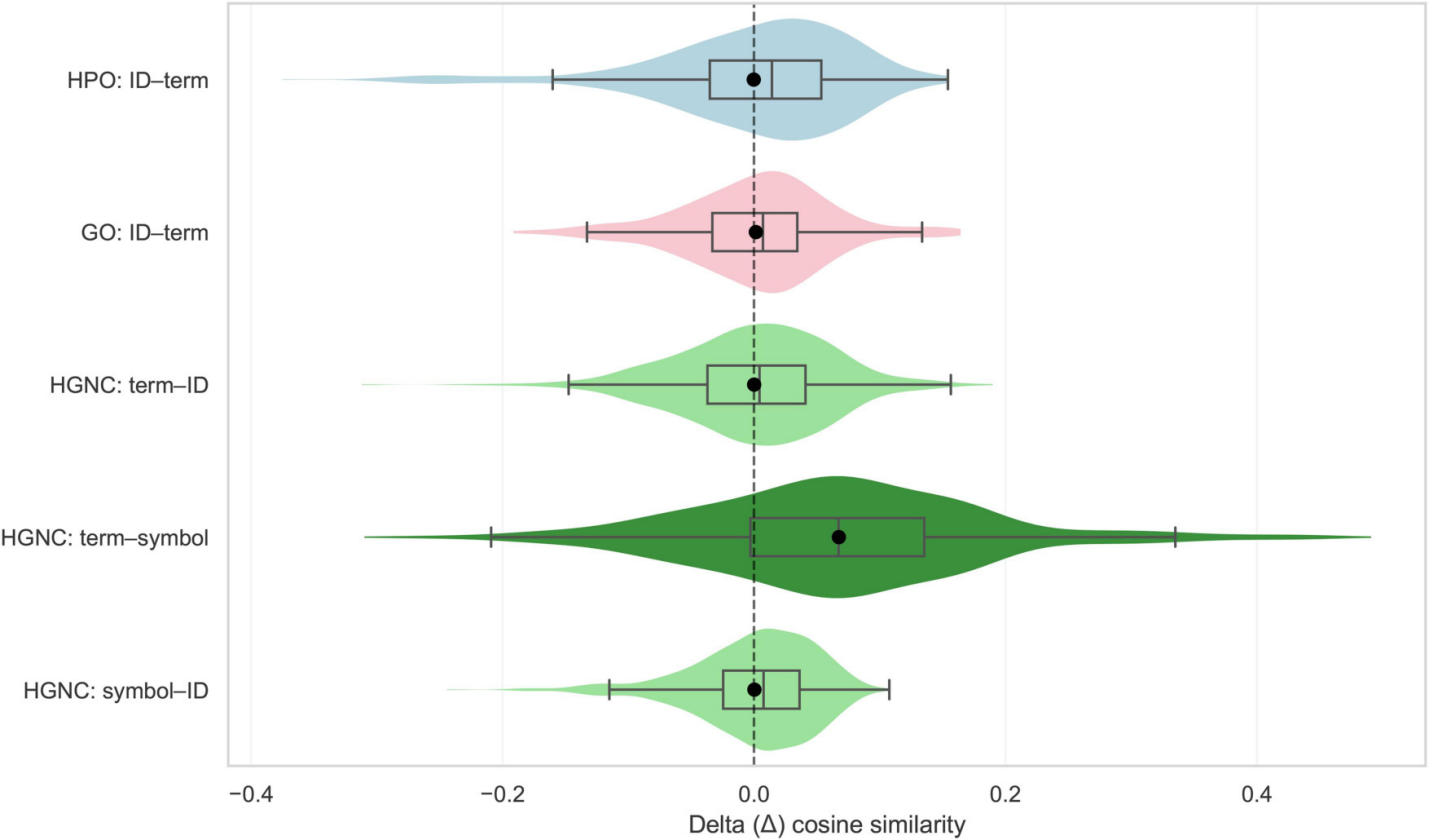
Hybrid violin–box plots showing the distribution of Δ cosine similarity for five terminology mappings. Δ is defined as the cosine similarity between a target term and its corresponding identifier (or gene symbol) minus the mean cosine similarity between the target term and all non-matching identifiers. Values near Δ=0 indicate no lexical association between terms and identifiers in the embedding space. HPO and GO term–ID pairs (light blue and light red) show Δ values centered near zero, indicating that these identifiers are not lexicalized in the model. Similarly, HGNC gene term–ID and symbol–ID pairs (light green) show little lexical association. In contrast, gene term–symbol pairs (dark green) display a higher Δ distribution, indicating that gene symbols share semantic similarity with gene terms. Tukey HSD testing confirms that the gene term–symbol distribution differs significantly from the other mappings.

Cosine similarity analyses provided convergent evidence (Figure 4). For each terminology, we computed the delta similarity Δ between matching term–identifier (or term–symbol) pairs and the mean similarity of all non-matching pairs. A one-way ANOVA on the five Δ distributions yielded a highly significant effect (F=90.95, $p=4.5\times 10^{-73}$), indicating systematic differences in semantic alignment across terminologies. Tukey post hoc comparisons showed that *only* the HGNC term–symbol pairs exhibited significantly elevated Δ values compared with all identifier-based mappings (HPO, GO, and HGNC), while all identifier-based mappings were statistically indistinguishable from one another. These findings demonstrate a robust lexicalization effect: gene symbols—unlike arbitrary identifiers—carry semantic information that aligns with the embeddings of their corresponding terms. Neither HPO nor GO identifiers, nor HGNC gene identifiers, show embedding-level evidence of semantic alignment. Thus, Llama’s pretrained representation space encodes meaningful similarity for lexicalized symbols but treats arbitrary identifiers as orthogonal token strings, explaining why only the HGNC term–symbol mapping supports generalization in downstream fine-tuning.

### Popularity

Descriptive statistics for three proxies of popularity are presented in Table 6. Mean values are shown for PubMed Central (PMC) identifier counts, annotation counts, and PMC term counts, grouped by whether the model correctly or incorrectly performed the term → identifier mapping task after fine-tuning. For both PMC identifier counts and annotation counts, correctly mapped terms exhibited higher values—indicating greater popularity—than incorrectly mapped terms. This relationship was less consistent for PMC term counts, suggesting that raw term frequency alone does not fully capture model familiarity with a term–identifier pair.

**Table 6:** Popularity proxies for seen terms.

Terminology	Annotation		ID		Term		
	Mean	SD	Mean	SD	Mean	SD	N
HPO (ID)							
Continuing correct	**308.3**	98.5	**10.7**	7.5	**360,215**	296,156	3
Gainer	**46.9**	101.6	**2.5**	5.4	**38,871**	104,958	124
Continuing incorrect	**7.0**	17.5	**0.6**	1.5	**23,105**	117,105	473
All HPO concepts	16.8	55.5	1.0	3.0	118,139		600
GO (ID)							
Continuing correct	**54.8**	58.0	**87.6**	86.5	**1,078,535**	1,332,578	5
Gainer	**27.8**	59.2	**15.0**	48.3	**73,530**	143,177	257
Continuing incorrect	**10.9**	27.9	**3.1**	8.7	**49,451**	115,829	337
All GO concepts	19.5	8.9	8.9	34.2	68,371	192,190	599
HGNC (symbol)							
Continuing correct	35.4	19.8	11,032.9	37,945.0	20,917	87,239	476
Gainer	31.7	20.7	2,791.9	3,685.1	11,143	30,499	117
Continuing incorrect	28.6	9.9	1,069.4	787.3	26,072	63,136	7
All HGNC symbols	34.6	19.9	9309.0	33,998.0	19,072	79,188	600
HGNC (ID)							
Gainer	**63.9**	65.7	1.4	2.6	23,555	33,267	7
Continuing incorrect	**34.3**	18.7	1.0	14.3	19,018	79,583	593
All HGNC identifiers	34.6	19.9	1.0	14.3	19,072	79,188	600

Means that are **bolded** differ within that group, one-way ANOVA, *p* < 0.01. HGNC gene symbols show orders-of-magnitude greater PMC exposure than HGNC numeric identifiers, consistent with their lexicalized use in biomedical text.

Pearson correlations among the three popularity proxies (computed on Laplace-smoothed, $\log_{10}$-transformed counts) are reported in Table 7. PMC identifier counts and annotation counts were moderately to strongly correlated for HPO (r=0.49) and GO (r=0.71), but only weakly correlated for HGNC numeric identifiers (r=0.16), reflecting the low and highly uniform PMC exposure of arbitrary HGNC identifiers. PMC term counts showed weaker and less consistent correlations with the other proxies across all three terminologies. Because PMC identifier counts most directly reflect the co-occurrence of a term–identifier pair in text likely to have been included in LLM pretraining corpora, they were used as the primary popularity proxy in subsequent analyses. Within HGNC, PMC symbol counts showed substantially stronger alignment with annotation counts (r=0.48) than PMC numeric identifier counts (r=0.16), consistent with the broader literature presence of lexicalized gene symbols relative to their arbitrary numeric counterparts.

**Table 7:** Pearson correlations among annotation counts and literature-based popularity proxies.

Terminology	Metric	Annotations	PMC term	PMC ID	PMC symbol
HPO	Annotations	–	0.399	0.494	–
	PMC term	0.399	–	0.257	–
	PMC ID	0.494	0.257	–	–
GO	Annotations	–	0.531	0.711	–
	PMC term	0.531	–	0.533	–
	PMC ID	0.711	0.533	–	–
HGNC	Annotations	–	0.189	0.161	0.481
	PMC term	0.189	–	0.152	0.296
	PMC ID	0.161	0.152	–	0.255
	PMC symbol	0.481	0.296	0.255	–

## Discussion

This study evaluated how well large language models learn biomedical term–code mappings across three representative terminologies (HPO, GO, and HGNC) using three base models (Llama 3.1 8B, Llama 3.1 70B, and GPT-4o) and multiple fine-tuned variants of Llama 3.1 8B. Four consistent patterns emerged. First, *model scale* strongly influenced baseline accuracy: larger pretrained models consistently performed better than smaller ones. Second, a robust *directionality* asymmetry was observed: term → identifier mappings were more accurate than identifier → term mappings across all terminologies. Third, fine-tuning produced heterogeneous *memorization* gains in the forward direction, with larger gains for GO than HPO and still smaller gains for mapping HGNC term → ID. *Generalization* to unseen (withheld) term–code pairs was largely confined to HGNC term → symbol mappings that involved lexicalized gene symbols.

### Model scale effects

Across tasks, GPT-4o achieved the highest baseline accuracy, followed by Llama 3.1 70B and Llama 3.1 8B (Table 3). Fine-tuning improved the 8B model relative to its own baseline. This pattern is consistent with scaling laws in which larger models benefit from greater representational capacity and broader pretraining exposure [6, 46, 47]. In some cases (Table 3), fine-tuning was able to close the gap between smaller models and larger ones.

### Directionality effects

A striking and consistent result was that term → identifier mapping outperformed identifier → term mapping for all models and terminologies (Table 4). We suggest that this asymmetry reflects the autoregressive training objective and the distributional structure of biomedical text. In the literature, natural language terms typically occur before their codes (e.g., *ataxia* followed by HP:0001251), so predicting an identifier given its preceding term is more aligned with common contexts encountered during pretraining than predicting a term given an identifier. This interpretation is consistent with directional failures described as the *reversal curse* [20], in which models trained on *A is B* do not reliably infer *B is A*. In the normalization setting, identifiers behave less like flexible semantic units and more like position-sensitive token sequences whose predictability depends on context order established during training.

### Popularity and factual salience

Fine-tuning outcomes also depended strongly on *popularity*: the likelihood that a term–code pair (or its components) was encountered during pretraining. Fine-tuned accuracy was highest for HGNC term → symbol mappings, intermediate for GO and HPO term → ID mappings, and lowest for mappings involving HGNC gene IDs (Table 5). This ordering parallels our popularity proxies (Table 6), suggesting that pretraining exposure influences the *factual salience* of mappings within model parameters. Prior work has shown that repeated exposure during pretraining strengthens a fact’s representational footprint and improves retrievability [48]. Conversely, long-tail facts with low exposure are weakly encoded and remain difficult to retrieve or consolidate through fine-tuning. A practical implication is that fine-tuning is most effective in a *reactive middle* of the frequency distribution. Very rare mappings may be too weakly represented to consolidate efficiently, whereas very common mappings may exhibit ceiling effects that limit measurable gains [18]. This helps reconcile why some tasks show large apparent improvements while others show modest gains, even with substantial fine-tuning.

Among the three popularity proxies, PMC identifier counts showed the strongest and most consistent correlations with annotation counts across terminologies (HPO: r=0.49; GO: r=0.71 (Table 7), whereas PMC term counts showed weaker and less consistent associations. This pattern supports using identifier co-occurrence frequency—rather than raw term frequency—as the primary proxy for pretraining exposure, because identifier strings are unambiguous markers of the specific term–identifier association that the model must memorize.

### Lexicalization and generalization

Popularity alone does not explain why generalization to unseen mappings occurred primarily for HGNC gene symbols. We propose that *lexicalization* is an additional enabling factor. HGNC gene symbols (e.g., TP53) are short, mnemonic strings that behave like meaningful tokens in biomedical text; in contrast, GO, HPO, and HGNC gene identifiers are intentionally arbitrary (e.g., HP:0001250). We define an identifier or symbol as *lexicalized* when its embedding is semantically aligned with the embedding of its associated term (or name), allowing the model to rely more on semantic structure and less on purely memorized token sequences when performing mapping operations.

Our embedding analyses are consistent with this mechanism. HGNC term–symbol pairs show higher within-pair cosine similarity than randomly paired terms and symbols, and PCA projections indicate partial overlap between gene names and gene symbols in embedding space (Figures 3, 4). However, the observed group differences in cosine similarity were modest, and lexicalization should be interpreted as a contributing factor rather than a dominant predictor in this dataset. By contrast, GO, HPO, and HGNC gene identifiers remain separated from their corresponding terms, consistent with a lack of semantic alignment. Under this view, fine-tuning on lexicalized symbols can improve performance on trained examples while also enabling some generalization to unseen pairs by leveraging pre-existing semantic structure, whereas fine-tuning on arbitrary identifiers is constrained largely to memorization.

### Interaction of popularity and lexicalization

Popularity and lexicalization are best understood as interacting rather than independent effects. Popularity increases the probability that a symbol or identifier co-occurs with descriptive context during pretraining; repeated co-occurrence can strengthen token associations and, for some symbol systems, promote semantic alignment in embedding space. Lexicalization, however, is primarily inherited from pretraining rather than created by fine-tuning: fine-tuning can amplify or *unlock* associations that are already supported by semantic structure, but it cannot easily endow arbitrary codes with meaning. This interaction yields a practical continuum: low-popularity, non-lexicalized identifiers are difficult to memorize; moderately popular but non-lexicalized identifiers can be memorized but do not generalize; and highly popular, lexicalized symbols support both memorization and (limited) generalization.

### Knowledge degradation

Knowledge gains were not cost-free. In several settings, fine-tuning degraded performance on some previously correct mappings, consistent with prior reports of knowledge degradation during fact injection [17]. This trade-off suggests that fine-tuning may overwrite or destabilize existing associations while strengthening others, a phenomenon related to catastrophic forgetting [49, 50]. Designing fine-tuning procedures that preserve prior knowledge while improving targeted mappings remains an important practical challenge.

### Term–identifier normalization and ontology learning

Term–identifier normalization can be situated within the broader framework of ontology learning (OL) by large language models [51], specifically as an instance of concept grounding: linking a natural-language label to its formal symbolic identifier within a single ontology. Unlike OL tasks such as taxonomy discovery or relation extraction, which operate over semantic relationships between concept labels, term–identifier normalization is largely memory-dependent when identifiers are weakly lexicalized. Identifiers such as HP:0001337 or GO:0005634 contain minimal semantic signal accessible to the model beyond ontology prefixes, limiting opportunities for inference based on semantic similarity. In such cases, correct output depends primarily on reproducing a specific token sequence. This differs from ontology matching (OM) tasks such as those addressed by OLaLa [52] and related work [53], where semantically equivalent labels are aligned across ontologies and both sides of the mapping retain lexical structure that can be exploited by similarity judgments. Our findings also engage a central question in the OL literature: to what extent LLMs rely on semantic structure vs. lexical priors acquired during pretraining. Mai et al. [51] showed that off-the-shelf LLMs fail on OL tasks when input labels are replaced with arbitrary strings, suggesting reliance on pre-existing lexical semantics. Our results offer a complementary perspective focused on the output side of the mapping. When the target identifier is arbitrary and weakly lexicalized, fine-tuning primarily reinforces memorization, with limited evidence of generalization to unseen term–identifier pairs. In contrast, when the target is lexicalized (e.g., HGNC gene symbols) and more closely aligned with the input term in embedding space, fine-tuning supports modest generalization consistent with leveraging existing semantic structure. Taken together, these findings suggest that lexical structure on both the input and output sides of a mapping is an important condition for LLMs to move beyond memorization in ontology-related tasks.

### Limitations

This study has several limitations. First, we operationalized memorization as gains on trained (seen) terms and generalization as correct predictions on withheld (unseen) term–code pairs; alternative definitions of generalization for factual fine-tuning may yield different interpretations. Second, our popularity measures rely on corpus proxies (e.g., PubMed Central counts and ontology annotation counts) rather than true pretraining statistics, which are unavailable. Third, embedding analyses were performed on the base Llama 3.1 8B model; how fine-tuning alters embedding geometry vs. reweighting token probabilities was not directly tested. Fourth, we did not evaluate cross-directional generalization (i.e., does term→identifier generalize to identifier→term) when fine-tuning exclusively on forward mappings. Given the autoregressive nature of LLMs and prior findings on directional asymmetry [20], such generalization is not expected and warrants explicit investigation in future work. Finally, we used a single PEFT setup (LoRA on Llama 3.1 8B) with fixed hyperparameters; other adapters, data mixtures, or larger fine-tuned backbones may change the memorization–generalization–degradation trade-offs.

### Future work

We acknowledge that the ontology task of binding an ontology concept to an arbitrary identifier is an especially difficult task for LLMs [4] and that due to long-tail effects fine-tuning is unlikely to emerge as the method for remedying this deficiency [3]. In real world settings, to be useful, LLMs must be able to retrieve the correct ID for each ontology term with near 100% accuracy to be usable. Retrieval augmented generation rather than fine-tuning is emerging as the dominant method to inject knowledge of ontology term-identifier pairs into LLMs [13, 14]. This reasonably raises the question as to whether it worthwhile to study acquisition of facts about ontology term-identifier pairs at all. We argue it is still worthwhile to experiment on how LLMs can be trained to learn facts about ontology term-identifier pairs because it offers insights into the importance of term and identifier popularity and term and identifier lexicalization as factors that control fact acquisition. These experiments offer insights into why some facts are learnable and others resist learning. Future work could provide insights into the importance of popularity and lexicalization in learning term-identifier pairs for other widely used medical ontologies and terminologies such as SNOMED CT, LOINC, and ICD.

## Conclusion

This study evaluated the capacity of large language models to normalize biomedical terms across three terminologies: HPO, GO, and HGNC-approved gene nomenclature. Using Llama 3.1 (8B and 70B) and GPT-4o as base models, together with several fine-tuned Llama 3.1 8B models, several consistent patterns emerged. Fact popularity—operationalized as the frequency of identifier strings and term–identifier co-occurrence in biomedical corpora—was strongly associated with memorization during fine-tuning. In this framework, popularity contributes to *factual salience*, the strength with which a mapping is represented in model parameters. GO identifiers, which appear more often in biomedical text than HPO identifiers, supported stronger memorization after fine-tuning, whereas less frequent HPO identifiers showed more modest gains.

HGNC provides a particularly instructive contrast because each gene is associated with both an arbitrary numeric identifier and a lexicalized gene symbol. In biomedical prose, gene symbols are widely used, whereas HGNC numeric identifiers are rarely written explicitly. Consistent with this distinction, lexicalized gene symbols showed stronger alignment with curated annotation counts and were more readily acquired during fine-tuning, whereas low-exposure numeric HGNC identifiers remained comparatively difficult to learn.

Lexicalization also appeared to shape generalization during fine-tuning. When terms and identifiers are more closely aligned in the model’s embedding space—most notably for gene names and gene symbols—the model is more likely to extend improvements beyond the fine-tuning set. By contrast, weaker semantic alignment between HGNC gene names and HGNC identifiers, between HPO terms and HPO identifiers, and between GO terms and GO identifiers was accompanied by limited generalization to withheld term–identifier pairs. Directionality and scale effects were consistent across settings. Term → identifier mapping generally outperformed identifier → term mapping, consistent with the autoregressive bias of large language models and the common presentation of ontology terms preceding identifiers in biomedical text. Larger base models consistently outperformed smaller ones. Finally, knowledge gains came with trade-offs: fine-tuning improved performance on seen terms and, in some cases, on withheld terms, but also degraded some preexisting knowledge—most noticeably for HGNC term-to-symbol mappings, where baseline accuracy was already near ceiling.

Together, these findings suggest that fine-tuning preferentially drives memorization when identifiers are common in the training corpus, and that generalization is most achievable when identifiers are also lexicalized. This *popularity–lexicalization continuum* provides a practical framework for predicting when fine-tuning will succeed, when it will fail, and why some terminologies remain resistant to robust acquisition of identifier-level facts.

## Data Availability

The original contributions presented in the study are included in the article/Supplementary Material, further inquiries can be directed to the corresponding author.

## Footnotes

^1^ https://github.com/clslabMSU/Finetuning-LLMs-for-Biomedical-Ontologies-Bidirectional-Mapping/tree/main/Datasets
